# The effects of time delays on synchronization properties in a network of neural mass models

**DOI:** 10.1186/1471-2202-14-S1-P78

**Published:** 2013-07-08

**Authors:** Robert Ton, Gustavo Deco, Andreas Daffertshofer

**Affiliations:** 1 Research Institute MOVE, VU University, Amsterdam, 1081 BT, The Netherlands; 2 Theoretical and Computational Neuroscience Group, Center for Brain and Cognition, Universitat Pompeu Fabra, Barcelona, 08018, Spain

## 

Patterns of (de)synchronization are key to neural functioning. We asked what general mechanisms are responsible for the emergence and disappearance of these (de)synchronization patterns. To answer this question we analytically revealed potential effects of physiologically motivated time delays in neural populations. We investigated a network of Neural Mass Models (NMM), here Freeman's seminal model that has successfully been used to model alpha-oscillations [1] and EEG activity in the olfactory system [2] and visual cortex [3]. In contrast to these local applications we were interested in distributed activities over larger networks and hence coupled arbitrarily many NMMs involving the aforementioned time delays. That is, we represented activity in distinct brain areas by means of oscillatory excitatory/inhibitory pairs and consider network behavior as representative for global brain activity.

We characterized synchronization as the appearance of clustered stationary phase probability density functions (PDFs). In general, this requires a phase dynamics description of the network, which can be established by applying a *slowly varying amplitude approximation *(see also [4]), together with a continuity equation covering the PDF's evolution. In fact we succeeded to show the existence of synchronized phase PDFs even for small inter area coupling strengths in the case of both zero and constant finite delays, whereas synchronization disappeared when delays were distributed throughout the network. Homogeneity of the effective coupling matrices (*D*) appearing in the phase dynamics equation is crucial in this respect as it is required for the existence of clustered phase PDFs. Loss of homogeneity in *D *can be caused by distributed delays as well as heterogeneity in the structural coupling matrix, leading to the conclusion that the mechanism modulating desynchronization is equal for both cases.

The effect of distributed delays is consistent with analytic results in phase oscillator systems [5,6]. This also holds for the drop in oscillation frequency for increased time delay values [5,7] that we observed in simulation results. These two findings validate the description of network behavior in terms of its phase dynamics.

In biologically more realistic networks time delays have been shown to determine network synchronization properties in a similar way [8]. Furthermore delays have turned out to be crucial in the behavior of resting state networks [9]. Our results suggest that structural heterogeneity may be compensated for by an appropriate distribution of time delays, thereby explaining the necessity of time delays in RSNs to establish biological plausible synchronization patterns.
